# A Plea for the Disappointed

**Published:** 1890-02-08

**Authors:** 


### The Doctor's Pulpit.

### A PLEA FOR THE DISAPPOINTED.

Who will find the man anywhere in the world who does not like to ride a winning horse? Women are probably more eager even than men to sit in the front rows at the theatre, and to receive the sacrament first at church. Not only does a man applaud himself when he comes in first at the end of the race, but all the bystanders and the great world that lies beyond conspire to give him the palm and the crown and the loud "huzzah." It is not to be wondered at, therefore, that we human creatures, being what we are, should fall down and worship strength and success, whilst we pity and despise, or despise without pitying, weakness and the want of success.

But conspicuous success belongs to the few; to the many appertains at best mediocrity, which in vast numbers of cases would more properly be called "failure." How many illustrious names does English literature present to the world? Yet what tens of thousands of ambitious, industrious, and not unworthy writers have offered life and soul on the altar of literary reputation from the comparatively recent days of Johnson and Goldsmith until now! The famous physicians and surgeons that English medicine can boast of, even from the earliest dawn of its history, may almost be numbered on the fingers of the two hands. And yet hundreds of medical men have died and will die in consequence of their strenuous and unceasing efforts to win name and fame in their special work. Are all the toilers who never rise to distinction to be classed with the multitudinous crowd of whom the conspicuous few think no more than they do of the rooks in a vast rookery, or of the ears of wheat in a limitless American clearing? Is there nothing to be said for the great unknown and their work? Was the world made for the famous few, and were the many intended as a mere background from which the successful ones might stand out in more pre-eminent relief?

It appears to the onlooker who strives to assume the attitude of disinterested observation, that vast numbers of the "unknown" certainly possess "gifts," but that they do not always make a discriminating use of them. They permit themselves to be carried away by the "rush" of the crowd, and instead of protesting on behalf of truth, justice, and reasonableness, they incontinently add their own voices to the swelling uproar of praise and adulation that acclaim the doings of the conspicuous few. Self-respect affirms that this is not only an unworthy, but a silly course to pursue. It is a kind of "carrying of coals to Newcastle," and a robbing of Penzance and Wick for that purpose. It is piling the praise and glory, which are already in excess, on the head of the conspicuous, and denying to the inconspicuous their just due. Too often it is the building up of a name which a wiser posterity will see ample reasons for levelling with the ground.

Let us enter a plea for the undistinguished many. Sunshine brightens the longest and most tedious journey. The sunshine of a just appreciation, of a discriminating judgment, of a generous sympathy—these are invaluable to the honest, the worthy, and the striving who have failed to command the attention of the world. In self-defence the inconspicuous are compelled to be philosophers. They ask themselves questions like these: Is life a racecourse, on which only one can be victorious, and all the rest are as worthless as a worn-out shoe? Or is it not rather a harvest-field, in which, though the mowers do the most heroic work, yet the humblest gleaner has his place, his duty, and his true value? Is it a prize-ring, in which hundreds play the part of mere spectators whilst two pound each other into miserable feebleness until one of them gives out and the other remains the sole victor? Or is it not rather a workshop, in which each man, woman, and child performs his allotted share of the work, and all unite in the production of the general result?

A phrase may kill, and a phrase may cure. Who knows the incalculable mischief that has been wrought, especially among English-speaking peoples, by the phrases, "Struggle for life," "Survival of the fittest," "Natural selection," and so on? There is no particular fault to be found with these formulæ from a scientific point of view. But their practical influence on everyday life has been in many cases most disastrous. They have been accepted for far more than they were ever worth. The strong have struggled more fiercely and less scrupulously for precedence because they have believed in the doctrine contained in these apparently obvious principles. The weak have laboured with less energy and hope, and have acquiesced with more tameness of spirit in the immediate results of the contest, because of their conviction that these same principles must for ever be true and undoubted.

The weak have been fools for their pains; and the strong have been equally fools in that they have too often bartered away their honour at the bidding of their strength. The world in its essence is not a race-course, nor a battle-field, nor a prize-fight. It is a place in which each man is given his own sphere to occupy and his own duty to do; and if that sphere be occupied honourably and that duty be done well, every man is undoubtedly a victor and a "fit survivor."

The general view of life which may prevail at any given moment is of the greatest possible interest to the practical physician. It is this, almost more than anything else, that makes or mars the health and happiness of an epoch. How can a man be well who, from morn till night, and from January to December, is incessantly exerting at the fullest tension every power of body, mind, and soul, in order to place himself at the head of all his competitors? And of the hundred who are always doing this, how can the ninety-nine who never reach the front place be other than desperate and miserable when their utmost efforts have failed? It is not in ordinary human nature to be either content or happy when its mind is firmly set on an object which it can never attain.

What, then, would the practical physician suggest who sees health destroyed and life made miserable by foolish and unequal contests? To advise the strong is of little use. They are wilful, defiant, indocile, and resolved at all risks to be at the head of their fellows, and nowhere else. This disposition is seldom associated with the highest moral worth; and therefore the highest moral arguments addressed to it are regarded with indifference, or even with disdain. But those who are less strong than the strongest, whose circumstances may be unfavourable, whose experience has often given them pause, and made them question both the wisdom and the expediency of continuing the contest on the present lines—they may listen to a word of reason from a fellow-toiler in the field of life.

This is the question: Is life to be regarded as a race in which only one or a few can win; or is it to be looked upon as a field to be wrought in, a forest to be cleared, a house to be built, in which the greatest and the smallest, and the smallest as well as the greatest, may each find his proper work and his fitting reward? Undoubtedly the plain sense of the plain man, the science of the scientist, and the religion of the most religious, will concur in this, that to make life one continual struggle for precedence and pre-eminence is the very madness of imbecility, and the very imbecility of madness. Can a pear turn itself into a plum; or a gooseberry into a peach? Can an Ethiopian become white like a Caucasian; or one of Stanley's Central African dwarfs reach the mental eminence of a Browning or a Tennyson? Parents are mad, schoolmasters are mad, college tutors are mad, successful men are mad when they exhort all youths indiscriminately to aim at the highest places in their several callings. Such a course is destitute of all reason, and leads to infinite immorality and misery. Instead of goading and urging everyone to aim at these highest places, to which by no possibility can the majority ever attain, let every boy and girl, every man and woman be taught to do his duty honestly, sturdily, and with his best powers day by day; and let such reward as that duty brings be considered all-sufficient. With this principle of life accepted, believed in, and faithfully lived by, health is possible, and happiness is possible. But whilst no man is content (or woman either) who does not occupy the first and foremost place in his own line of things, health and happiness are bound to go from bad to worse; and the only person who reaps any profit by it is the devil.

